# Dr. Wilbur S. Nutten

**Published:** 1904-05

**Authors:** 


Dr. Wilbur S. Nutten, of Newark, N. Y., died at his home April 18, 1904, aged 64 years. He graduated from the College of Physicians and Surgeons, New York, in 1863, and was a member of the various local and state medical societies.

